# Case report: Compound heterozygous mutations in the *KDSR* gene cause progressive keratodermia and thrombocytopenia

**DOI:** 10.3389/fped.2022.940618

**Published:** 2022-07-26

**Authors:** Li Wu, Yajie Zhang, Juan Zi, Yinyan Yan, Lihua Yu, Danna Lin, Lulu Huang, Xiaorong Lai, Xu Liao, Lihua Yang

**Affiliations:** Department of Pediatric Hematology, Zhujiang Hospital, Southern Medical University, Guangzhou, China

**Keywords:** *KDSR*, keratodermia, thrombocytopenia, children, case report

## Abstract

KDSR (3-ketodihydrosphingosine reductase) is a short-chain dehydrogenase located in the endoplasmic reticulum. Mutations in KDSR cause defects in ceramides, which play a key role in the biological processes of the skin and other tissues. Herein, we report a case of compound heterozygous mutations in *KDSR* that caused progressive keratodermia and thrombocytopenia in a 2-year-old male patient.

## Introduction

The *KDSR* gene encodes 3-ketone dihydrosphingosphingosine reductase, which is an essential enzyme in the early stages of sphingolipid synthesis (1, 2). Here, we report a case with mild thrombocytopenia and progressive keratodermia (perianal, palms, trunk and cheek skin keratosis disorders). The phenotypic characteristics of this patient, resemble that of other affected individuals described in the literature for which an autosomal recessive mode of inheritance have been proposed for pathogenic variants on the *KDSR* gene (3–10). Erythrokeratoderma with thrombocytopenia as a cause of autosomal recessive erythrokeratoderma is caused by mutations in the *KDSR* gene. This syndrome was described by Bursztejn et al. (8).

## Case presentation

The patient is a 2-year-old male. He was the first child born to unrelated healthy parents from China. He was delivered at 39 + 3 weeks by normal spontaneous vaginal birth and his birth weight was 3.5 kg. At birth, he was covered in thick adherent plate-like scales, but eclabium and ectropion were not observed. Then, the thick scales desquamated gradually over the first month of life.

At the age of 11 months, a blood count showed a mild, isolated thrombocytopenia (platelet count, 60–100 × 10^9^/L) during a regular physical exam. However, at the age of 14 months, the platelet count dropped to 35 × 10^9^/L. The bone marrow morphology test showed an increased number of megakaryocytes and dysplasia. A diagnosis of primary immune thrombocytopenia (ITP) was made. He was treated with ShengXuexiaoban capsules for several months and had a slight increase in platelets (50–120 × 10^9^/L). ShengXuexiaoban capsules are part of traditional Chinese medicine. They are composed of Natural Indigo, Weeping Forsythia Capsule, Hairyvein Agrimonia Herd, Tree peony Bark, Liquorice Root. ShengXuexiaoban capsules have been shown to be effective clearing away heat toxic materials, cooling blood and hemostasis, scattering stasis and eliminating spot.

At age 15 months, he was hospitalized because of “perianal hyperkeratosis”. The pathologic analysis revealed squamous hyperplasia, with obvious parakeratosis and dyskeratosis.

**Figure 1:**
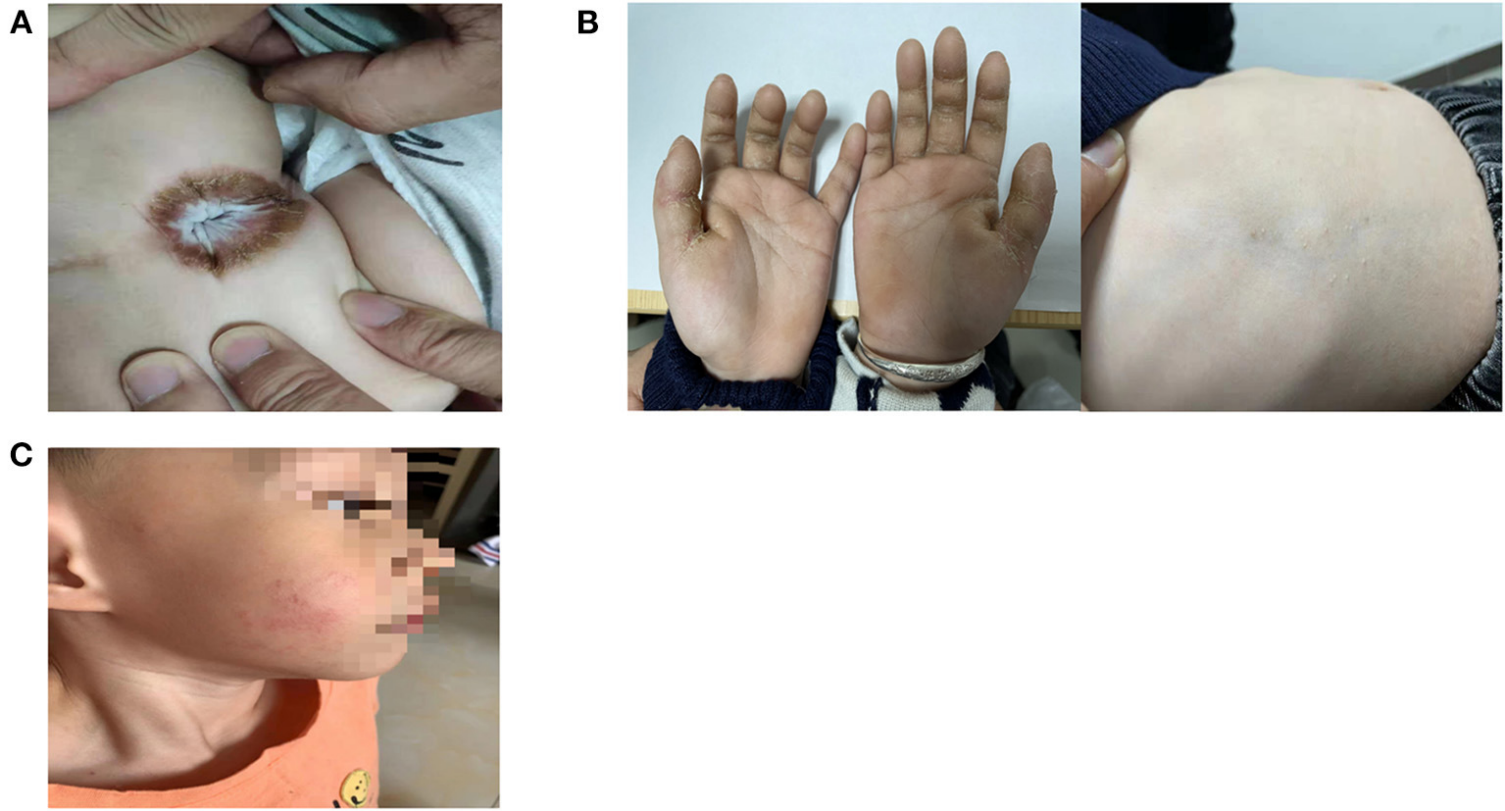
**(A)** Perianal hyperkeratosis. **(B)** Diffuse palmar keratoderma and limited trunk keratoderma. **(C)** Limited cheek keratoderma.

**Figure 2:**
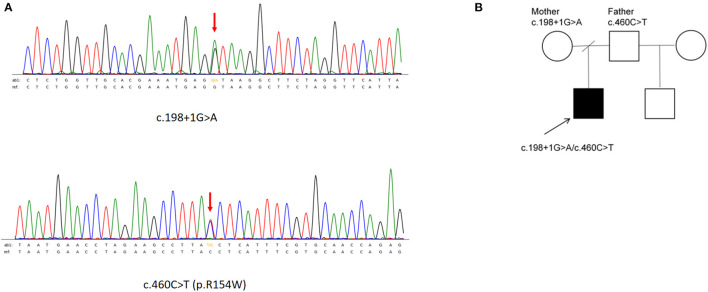
**(A)** The result of Sanger sequencing analysis. **(B)** Pedigree and variants identified in KDSR.

He presented to our hospital at the age of 2 years, and perianal hyperkeratosis was observed on examination (Figure 1A). The platelet count was 57 × 10^9^/L, and the peripheral lymphocyte subsets were both normal. Whole exome sequencing results showed compound heterozygous mutations in the *KDSR* gene (WES is performed according to the following methodology: Genomic DNA was extracted and fragmented. The paired-end libraries were prepared. Custom-designed NimbleGen SeqCap probes were used for in-solution hybridization to enrich target whole-exome sequences. Captured DNA samples were amplified by PCR. Sequences were aligned to the hg19 reference genome by NextGENe software.). WES results were confirmed by Sanger Sequencing analysis (Figure 2). As shown in the pedigree, the mother of the patient is a carrier of variant NM-002035 c.198 + 1G>A, while variant NM-002035: c.460C>T (p.R154W) was inherited from the paternal side of the family.

At the age of 2 years and 6 months, there were cracks in his hands and trunk and the appearance of hyperkeratosis (Figure 1B). Then, he was treated with a systemic retinoic acid derivative (1 mg/kg/d) and tretinoin cream for 2 months, but his skin lesions did not resolve. At the age of 3 years, his cheek had new lesions (Figure 1C). The platelet count was maintained at (60–85) × 10^9^/L, with no bleeding.

**Figure 3:**
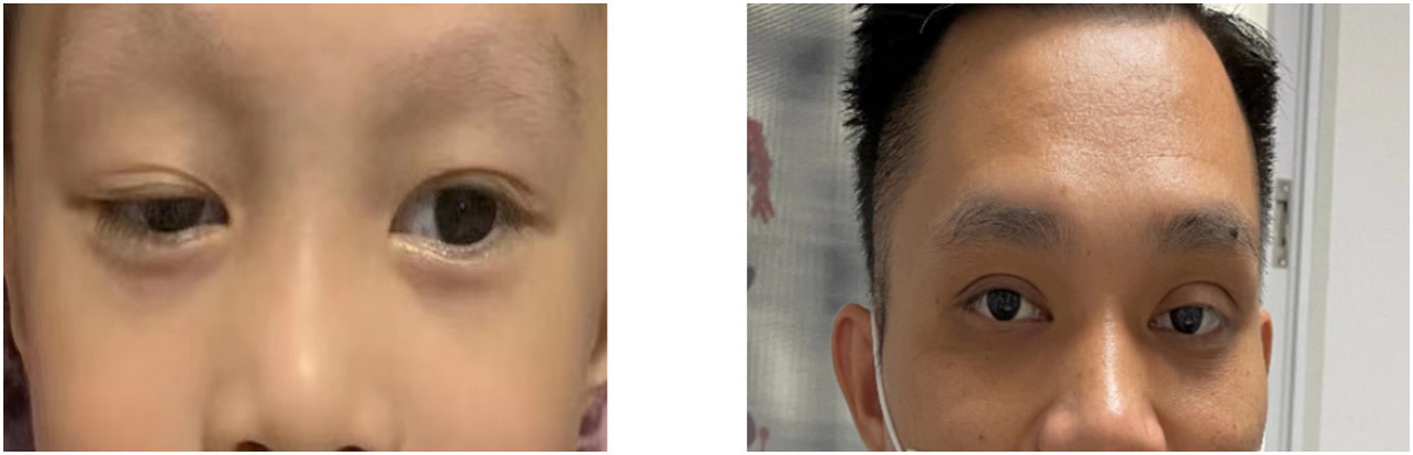
The patient suffered from ptosis of right eye, and his father suffered from ptosis of leftt eye (his father had upper lid blepharoppasty).

In addition, the patient suffered from ptosis of the right eye at birth, and his father and grandfather also presented with ptosis of the left eye (Figure 3). Whether the phenotype of ptosis is associated with KDSR mutations has not been reported in the literature.

**Figure 4:**
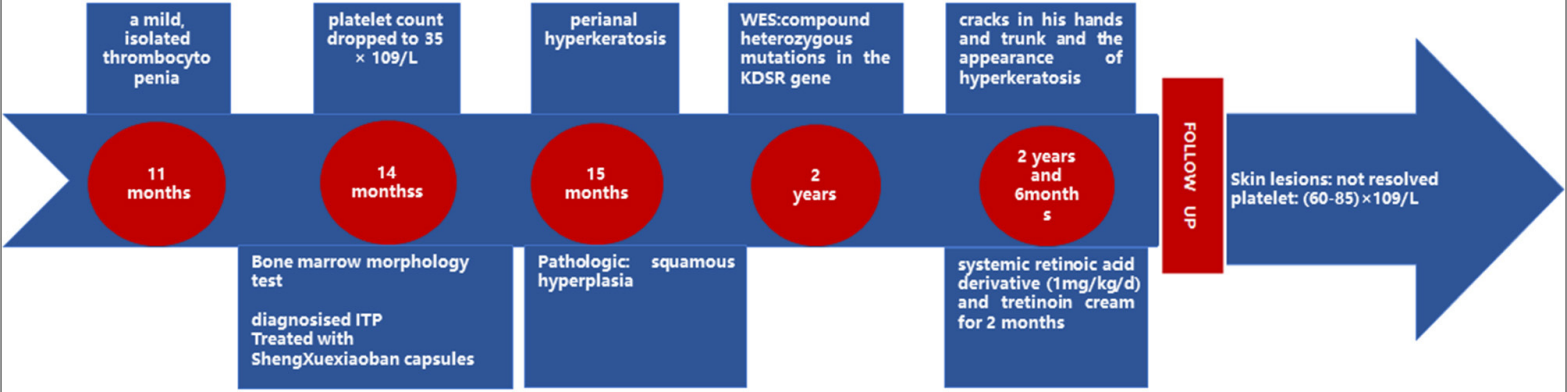
Case progress timeline.

Case Progress Timeline is shown in the Figure 4.

## Discussion

KDSR is a short-chain dehydrogenase located in the endoplasmic reticulum. It has a transmembrane domain at the N-terminus (amino acids 1-21) and two transmembrane domains at the C-terminus (amino acids 271-291 and 294-314). The active site in KDSR is found in the middle of the protein (amino acids 22-270). KDSR is the key essential enzyme in the ceramide *de novo* synthesis pathway (2). Mutations in *KDSR* cause defects to ceramides, which are vitally important. In addition to maintaining membrane structure integrity, ceramides are also essential for crucial signaling processes. In the clinical phenotype, mutations in *KDSR* can manifest as keratinization disorders, severe thrombocytopenia, anemia and hepatic angioendothelioma (3–10).

There are certain differences in the clinical phenotypes of keratosis caused by *KDSR* mutations, ranging from diffuse hyperkeratosis to localized keratodermas. Four patients with progressive symmetrical erythematosus (progressive symmetric erythrokeratoderma, PSEK) with *KDSR* compound heterozygous mutations were first reported by Boyden et al. (10). The face, palms, soles, and genitals were the most severely affected areas in all patients. The skin symptoms in patients receiving treatment with a tretinoin derivative (isoretinoin) were visibly resolved. Takeichi et al. subsequently reported 4 patients with compound heterozygous mutations in *KDSR*. Two of these patients were hyperkeratotic, limited to the palmar, plantar, and anal-genital skin, while the other two patients had more severe, harlequin ichthyosis-like skin. Treatment with isoretinoin did not work. In two patients with *KDSR* compound heterozygous mutations reported in Bariana et al. (7), there were only mild manifestations of minimal involvement in the skin. Bursztejn et al. (8) reported a case in which the skin lesions were mainly located in the face and perianal area. In addition to keratosis, there was also locally abnormal orange skin. The patient's serum carotene was twice the normal level.

The mechanism by which *KDSR* mutations cause skin lesions is not fully understood. Boyden et al. (10) found expansion of filaggrin (FLG) immunostaining in the affected tissue, and these results suggest that patients with *KDSR* mutations have defects in the terminal differentiation of keratinocytes. Takeichi et al. (9) showed a decrease in ceramide levels in skin biopsies of patients, while terminal differentiation markers, such as keratin 10, keratin 14 and filaggrin, were increased.

This finding supports the hypothesis that *KDSR* mutations lead to the dysregulation of ceramide biosynthesis and suggests that a decrease in KDSR activity leads to a decrease in ceramide levels in the skin. In the study of Pilz et al. (4), a patient with compound heterozygous mutations of *KDSR* had unusual keto-type ceramides in the lesion areas. The formation of keto-type ceramides may be a bypass by ceramide synthases due to the limited function of the mutated KDSR enzyme in the metabolic pathway. Bypass products of keto-type ceramides are a possible cause of skin lesions.

In some patients, the skin symptoms were significantly resolved after oral isotretinoin treatment, but in our case, they were not. The reason why isotretinoin therapy is effective may be because it compensates for a genetic defect in the ceramide *de novo* synthesis pathway through alternative pathways of drug-induced ceramide production (10).

Patients with *KDSR* mutations may present with severe thrombocytopenia. In Takeichi et al., Bariana et al., and Liu et al. (6, 7, 9), the platelet count remained low (−20 × 10^9^/L) with recurrent skin, rectal, and gingival bleeding. In our case, the platelet count was maintained at (60–85) × 10^9^/L, and there were no bleeding manifestations.

Defects in platelet formation and release in the final stage of thrombopoiesis may be the primary cause of thrombocytopenia in patients with *KDSR* mutations (9). Bariana et al. (7) reproduced thrombocytopenia by knocking out the *KDSR* gene in zebrafish, and the abnormal morphology and function of the patient's megakaryocytes was rescued *in vitro* by reprogramming the bone marrow pluripotent stem cells of patients with mutations in the K*DSR* gene. The key role of KDSR in platelet formation was further confirmed. Therefore, when patients with *KDSR* mutations have repeated bleeding due to thrombocytopenia, which is life-threatening, hematopoietic stem cell transplantation should be considered.

*KDSR* mutations may cause other rare phenotypes. Liu et al. reported an infant with homozygote *KDSR* mutations, and he was also diagnosed with hepatic hemangioendothelioma at birth (6). In current animal models, *KDSR* mutations induce liver damage in zebrafish. Researchers believe that genetic mutations that cause reduced activity in KDSR may be potential risk factors for the development of liver disease, and patients with these mutations may be highly susceptible to steatohepatitis, fibrosis, or hepatocellular carcinoma (11). In our case, the patient suffered from ptosis of the right eye, and his father and grandfather suffered from ptosis of the left eye. Whether this phenotype is related to KDSR mutations needs more cases and studies to verify such an association.

In addition, a new mutation site of *KDSR*, c.198 + 1G>A, was found in this case. The mutation is located in the splicing region, and its sequence is highly conserved. This mutation is not currently reported in the population gene pool and a variety of computer-aided algorithms have predicted that this change may affect protein function. According to ACMG criteria, this variant is classified as likely pathogenic [Pathogenicity analysis evidence composition: (PVS1_M+PM2+PM3+PP4)].

## Conclusion

In conclusion, our data introduce a novel pathogenic mutation of *KDSR* that is associated with cutaneous keratosis and mild thrombocytopenia. In addition, we found unilateral eyelid drooping may be rare phenotype of *KDSR* mutations. A careful watch-and-wait approach rather than early intervention may be more appropriate in patients with *KDSR* mutations, unless there is a risk of life-threatening bleeding.

## Data availability statement

The original contributions presented in the study are included in the article/supplementary material, further inquiries can be directed to the corresponding authors.

## Ethics statement

The studies involving human participants were reviewed and approved by Medical Ethics Committee of Zhujiang Hospital of Southern Medical University. Written informed consent to participate in this study was provided by the participants' legal guardian/next of kin. Written informed consent was obtained from the individual(s), and minor(s)' legal guardian/next of kin, for the publication of any potentially identifiable images or data included in this article.

## Author contributions

LW drafted the manuscript. YZ, JZ, YY, LYu, DL, LH, XLa, and XLi collected materials and prepared figures. LYa critically revised the final manuscript. All authors contributed to the study and approved the final submitted version of the manuscript.

## Conflict of interest

The authors declare that the research was conducted in the absence of any commercial or financial relationships that could be construed as a potential conflict of interest.

## Publisher's note

All claims expressed in this article are solely those of the authors and do not necessarily represent those of their affiliated organizations, or those of the publisher, the editors and the reviewers. Any product that may be evaluated in this article, or claim that may be made by its manufacturer, is not guaranteed or endorsed by the publisher.
